# *In vitro* characterization of six *STUB1* variants in spinocerebellar ataxia 16 reveals altered structural properties for the encoded CHIP proteins

**DOI:** 10.1042/BSR20170251

**Published:** 2017-04-28

**Authors:** Yasaman Pakdaman, Monica Sanchez-Guixé, Rune Kleppe, Sigrid Erdal, Helene J. Bustad, Lise Bjørkhaug, Kristoffer Haugarvoll, Charalampos Tzoulis, Ketil Heimdal, Per M. Knappskog, Stefan Johansson, Ingvild Aukrust

**Affiliations:** 1 Center for Medical Genetics and Molecular Medicine, Haukeland University Hospital, Bergen, Norway; 2 Department of Clinical Science, University of Bergen, Bergen, Norway; 3 K.G. Jebsen Centre for Neuropsychiatric Disorders, Department of Biomedicine, University of Bergen, Bergen, Norway; 4 Department of Biomedicine, University of Bergen, Bergen, Norway; 5 Department of Biomedical Laboratory Sciences and Chemical Engineering, Western Norway University of Applied Sciences, Bergen, Norway; 6 Department of Neurology, Haukeland University Hospital, Bergen, Norway; 7 Department of Clinical Medicine, University of Bergen, Bergen, Norway; 8 Department of Medical Genetics, Oslo University Hospital, Oslo, Norway; 9 K.G. Jebsen Centre for Neuropsychiatric Disorders, Department of Clinical Science, University of Bergen, Bergen, Norway

**Keywords:** CHIP, co-chaperone, protein aggregation, protein misfolding, ubiquitin ligases

## Abstract

Spinocerebellar ataxia, autosomal recessive 16 (SCAR16) is caused by biallelic mutations in the STIP1 homology and U-box containing protein 1 (*STUB1*) gene encoding the ubiquitin E3 ligase and dimeric co-chaperone C-terminus of Hsc70-interacting protein (CHIP). It has been proposed that the disease mechanism is related to CHIP’s impaired E3 ubiquitin ligase properties and/or interaction with its chaperones. However, there is limited knowledge on how these mutations affect the stability, folding, and protein structure of CHIP itself. To gain further insight, six previously reported pathogenic *STUB1* variants (E28K, N65S, K145Q, M211I, S236T, and T246M) were expressed as recombinant proteins and studied using limited proteolysis, size-exclusion chromatography (SEC), and circular dichroism (CD). Our results reveal that N65S shows increased CHIP dimerization, higher levels of α-helical content, and decreased degradation rate compared with wild-type (WT) CHIP. By contrast, T246M demonstrates a strong tendency for aggregation, a more flexible protein structure, decreased levels of α-helical structures, and increased degradation rate compared with WT CHIP. E28K, K145Q, M211I, and S236T also show defects on structural properties compared with WT CHIP, although less profound than what observed for N65S and T246M. In conclusion, our results illustrate that some *STUB1* mutations known to cause recessive SCAR16 have a profound impact on the protein structure, stability, and ability of CHIP to dimerize *in vitro.* These results add to the growing understanding on the mechanisms behind the disorder.

## Introduction

The recessively inherited group of cerebellar ataxias (autosomal recessive cerebellar ataxia, ARCA) is commonly characterized by early onset and gradual worsening of gait, balance, and coordination over months and years. Friedreich ataxia (prevalence of 2–4/100,000) is known to be the most frequent type of ARCA, followed by ataxia telangiectasia (prevalence 1–2/100,000) and early onset cerebellar ataxia with retained tendon reflexes (prevalence 1/100,000) [1,2]. Many new ARCA genes have been identified recently due to the use of whole exome sequencing (WES) as routine in several diagnostics laboratories. Recently, it was shown that recessive mutations in *STUB1* can cause spinocerebellar ataxia, autosomal recessive 16 (SCAR16) in some families [3–10].

*STUB1* encodes STUB1 (STIP1 homology and U-box containing protein 1), also known as C-terminus of Hsc70-interacting protein (CHIP), an evolutionary conserved protein of ∼35 kDa that is highly expressed as a dimeric co-chaperone in tissues that are active in terms of metabolism and protein turnover, such as brain, heart, and skeletal muscle. The protein was first identified as a tetratricopeptide repeat (TPR) domain-containing protein during a human cDNA library screening looking for proteins with a TPR domain possibly involved in stress regulation [11]. Further structural analysis revealed similarities between the C-terminus of CHIP (the U-box) and the E3 ligase component of the ubiquitin–proteasome pathway, suggesting an active role in ubiquitination of chaperone substrates for CHIP [12]. Thus, CHIP was discovered as the first ubiquitin ligase that directly associates with molecular chaperones. CHIP labels non-native proteins unable to be refolded by chaperones for proteasomal degradation [12–14].

The primary structure of CHIP has two main domains: an N-terminal TPR domain mediating the interaction of CHIP with Hsp70 and Hsp90 molecular chaperones; and a C-terminal U-box domain facilitating ubiquitination of chaperone substrates through the interaction with different E2 enzymes. These domains are separated by a central helical hairpin region (also termed the central coiled-coil (CC) domain), which influences the dimerization and stability of the whole protein [15].

**Figure 1:**
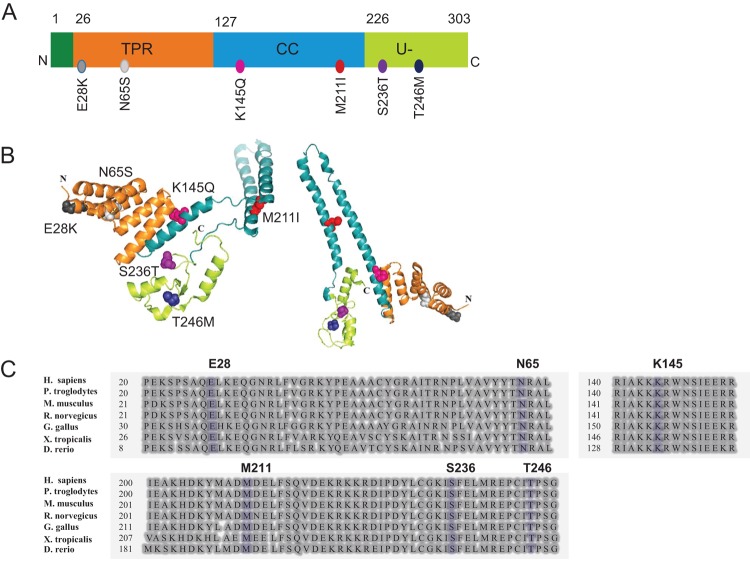
The position of the six selected variants in the CHIP protein The position of selected variants is shown in (**A**) protein 1D structure and (**B**) 3D protein structure of the dimeric CHIP with colorful spheres. The 3D protein structure was created by using PyMOL software (https://www.pymol.org) [Protein Data Bank (PDB) code: 2C2L]. Minor modifications were performed in order to separate the two monomers of the dimer and to better visualize the location of each mutation as well as different protein domains. (**C**) Alignment of CHIP protein sequences from human (*H. sapiens*), chimpanzee (*P. troglodytes*), mouse (*M. musculus*), rat (*R. norvegicus*), chicken (*G. gallus*), frog (*X. tropicalis*), and zebra fish (*D. rerio*). Selected variants are shown in blue. The alignment was performed using the NCBI HomoloGene tool (https://www.ncbi.nlm.nih.gov/homologene).

To date, 19 pathogenic *STUB1* variants have been described associated with SCAR16 according to the Human Gene Mutation Database [16]. Studies reporting the structural/functional consequences of these variants are few and the protein folding and stability properties of CHIP variants have so far been poorly investigated. Six *STUB1* variants, i.e. E28K, N65S, K145Q, M211I, S236T, and T246M were selected for the present study. The overall aim was to characterize the structural properties of these CHIP mutants with special focus on protein structure, stability, and CHIP’s ability to oligomerize *in vitro*, with the aim to discover new mechanisms for disease development of SCAR16. The six *STUB1* variants have been identified and reported as pathogenic in individuals with SCAR16 [3,5,7,10]; E28K and N65S were identified in two families with ARCA and cognitive impairment by our group [3]. T246M was first described by Shi et al. [9 in a patient with ataxia and hypogonadotropic hypogonadism. This variant was included in the study due to phenotypic similarity with patients carrying the E28K and N65S mutations. Variants K145Q, M211I, and S236T were additionally selected to represent variants from CHIP protein domains reported to be crucial for CHIP dimerization or protein–protein interactions [5,7,10]. All these variants affect residues that are conserved across eukaryotic species. The positions of these variants within the CHIP protein structure as well as their position within the amino acid sequence alignment are shown in Figure 1.

## Materials and methods

### Plasmids and mutagenesis

Full-length cDNA encoding human CHIP was subcloned into the bacterial expression vector pETM-41 (EMBL) as previously described [3]. All *STUB1* variants were made using QuikChange XL Site-directed Mutagenesis Kit (Agilent Technologies). We verified the sequence of all constructs by Sanger sequencing.

### Expression and purification of CHIP from *Escherichia coli*

The His-maltose-binding protein (MBP)-CHIP fusion proteins were expressed for 24 h at 25°C in the BL21-CodonPlus (DE3)-RP *E. coli* strain (Agilent Technologies). Cells were harvested by centrifugation at 4000×***g*** and lysed by sonication in 5 ml/g wet weight lysis buffer (50 mM NaH_2_PO_4_·H_2_O, 300 mM NaCl, 10 mM imidazole, 20 mM 2-mercaptoethanol, 0.1% Tween-20, pH 8). The His-MBP-tagged proteins were purified using Ni-NTA agarose nickel resin (Qiagen), according to manufacturer’s instruction. To generate CHIP, the His-MBP-tagged proteins were cleaved by Tobacco etch virus (TEV) protease (molar ratio 1:10) for 2 h at room temperature. In order to choose an appropriate buffer for protein storage and further experiments, TEV cleaved wild-type (WT) MBP-CHIP proteins were dissolved in three different buffer compositions and centrifuged for 19 h at 200000×***g*** (80000 rpm) at 4°C. All the pellets and supernatants were analyzed for CHIP protein content, using sodium dodecyl sulfate/polyacrylamide gel electrophoresis (SDS/PAGE) and followed by Coomassie Blue staining.

### CHIP ubiquitination activity assay

*In vitro* ubiquitination activity assay was set up for both MBP-fusion and MBP-cleaved CHIP recombinant proteins. The reactions were prepared in a total volume of 20 μl, containing 2.5 μM (MBP)-CHIP (E3), 2.5 μM UbcH5c (E2) (Boston Biochem), 0.05 μM Ube1 (E1) (Boston Biochem), 250 μM ubiquitin (Boston Biochem), and 0.8 μM recombinant human His-HSPA8 (HSC71) (CHIP substrate) (Life Technologies AS, Thermo Fisher Scientific) in an ubiquitination buffer (50 mM Tris HCl, pH 7.5, 0.6 mM DTT, 2.5 mM Mg-ATP) and incubated at 37°C for 1 h. Protein samples were analyzed by SDS/PAGE and immunoblotting using either anti-CHIP (LS-C137950, LifeSpan Bioscience) or anti-Hsc70 (ADI-SPA-815-F, Enzo Life Sciences) to monitor CHIP self-ubiquitination or Hsc70 ubiquitination respectively.

### Limited proteolysis assay

Trypsin was added to 30 μg of the MBP-cleaved CHIP protein at a CHIP to trypsin ratio of 1:600 (by mass) in a 100 μl reaction mixture (50 mM NaCl, 20 mM Hepes, and 2 mM DTT) and incubated at 25°C. The reactions were terminated at different time points (0, 5, 10, 20, and 30 min) by adding 18.5 μl of the reaction mixture to 5 μl NuPAGE LDS Sample Buffer (4×) (ThermoFisher Scientific), 1.4 μl NuPAGE Sample Reducing Agent (10×) (ThermoFisher Scientific), and 1 μl of the trypsin inhibitor (prepared at a trypsin to trypsin inhibitor mass ratio of 1:1.5). Samples were analyzed by SDS/PAGE followed by SYPRO Ruby staining and further quantified by Image Processing and Analysis in Java software (ImageJ, National Institutes of Health).

### Size-exclusion chromatography

Different oligomeric states of the WT and mutant MBP-CHIP fusion proteins were studied by gel-filtration, using a Superdex 200 Increase 10/300 GL column (GE Healthcare) on the BioLogic DuoFlowTM Medium-Pressure Chromatography System (Bio-Rad Laboratories). The column was first equilibrated overnight with phosphate buffer (20 mM NaH_2_P0_4_, pH 7.4). One milligram of MBP-CHIP protein diluted in running buffer was prepared by centrifugation (13000×***g***, 10 min) and gel filtrated at a constant flow rate of 0.3 ml/min. Protein fractions were collected and analyzed using SDS/PAGE and Coomassie Blue staining.

### Native polyacrylamide gel electrophoresis

Different conformational states of the WT and mutant CHIP proteins were studied by native gel electrophoresis and further analyzed by Coomassie Blue staining. Ten micrograms of MBP fusion protein (WT and mutants) was analyzed by NativePAGE^TM^ Bis-Tris gels (12%) (ThermoFisher Scientific) in Native PAGE^TM^ sample buffer using NativePAGE™ Anode Buffer (1×) and light blue NativePAGE™ Cathode Buffer (1×) as described by the manufacturer.

### Circular dichroism spectroscopy

All the samples were prepared at a protein concentration of 6 μM in a buffer containing 10 mM potassium phosphate (pH 7.4) and 100 mM sodium fluoride. Protein concentrations were determined spectrophotometrically by using the theoretical extinction coefficient at *A*_280_ of 1.224 M^−1^cm^−1^ for WT-CHIP (and mutants) and 0.512 M^−1^cm^−1^ for MBP. CD spectra were recorded at 20°C in a Jasco J-810 spectropolarimeter equipped with a Peltier temperature control unit (Jasco Products). The far-UV measurements were recorded from 185 to 260 nm using a light path of 1 mm and the spectra obtained were the average of four scans at a scan rate of 50 nm/min. The final spectra were presented after buffer subtraction. Thermal denaturation profiles were obtained by recording the decrease in ellipticity at 222 nm as a function of temperature in the range 20–90°C with a scan rate of 40°C/h.

## Results

### Expression and purification of WT and mutant forms of CHIP

**Figure 2:**
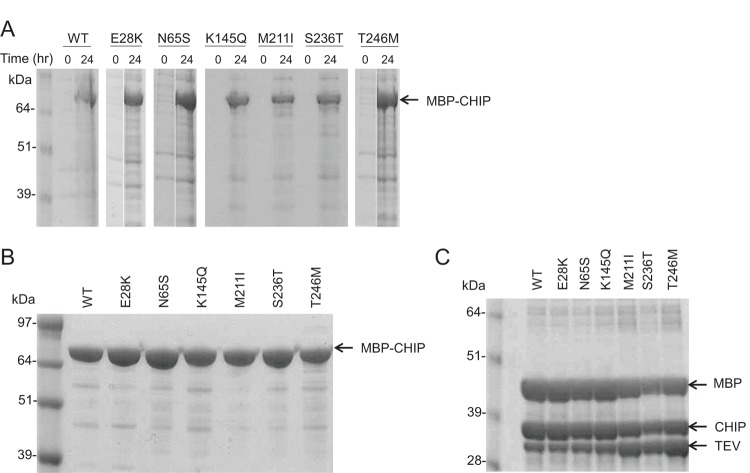
Expression and purification of WT and mutant forms of MBP-CHIP (**A**) The expression of MBP-CHIP proteins of both WT and mutant forms at time 0 and 24 h after induction of protein expression by 0.5 mM IPTG in BL21 *E. coli* cells. (**B**) Purified WT and mutant forms of MBP-CHIP after Ni-NTA purification procedure. (**C**) WT MBP-CHIP fusion protein cleavage, using TEV protease to MBP-CHIP mass ratio of 1:10 and an incubation time of 2 h. All samples were analyzed by SDS/PAGE (10% gel) and Coomassie Blue staining.

WT and mutant forms of MBP-fused CHIP were expressed in *E. coli*, and successful protein expression was verified for each mutant after IPTG induction as shown in Figure 2(A). Furthermore, fusion His-MBP-CHIP (WT and mutants) were purified by nickel resin (Figure 2B) and cleaved by TEV protease (Figure 2C). For minimal aggregation of CHIP recombinant protein, 100 mM HEPES (pH 8), 5 mM DTT, 100 mM NaCl, and 10% glycerol were selected as the storage buffer (Supplementary Figure S1).

### *In vitro* ubiquitination activity of WT and mutant forms of CHIP

**Figure 3:**
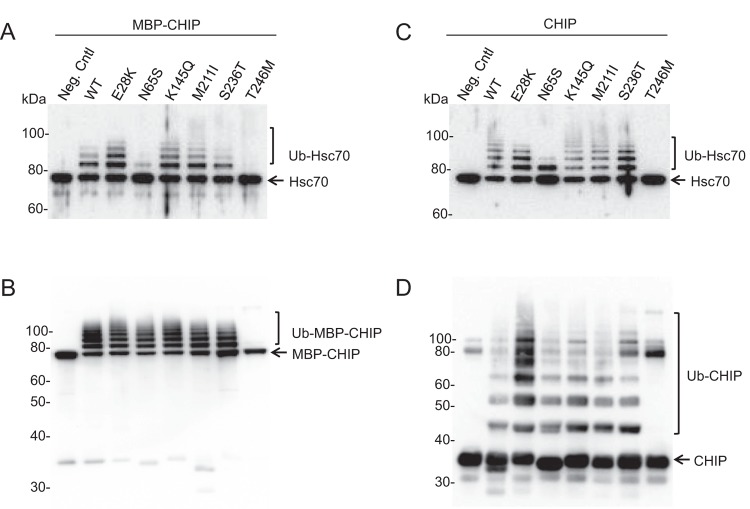
Ubiquitination activity of recombinant WT and mutant CHIP proteins *In vitro* ubiquitination activity assay was performed using WT and mutant CHIP as ubiquitin ligases and Hsc70 recombinant protein as substrate. Hsc70-ubiquitination (**A** and **C**) and self-ubiquitination activities (**B** and **D**) were explored on MBP-CHIP fusion proteins (**A** and **B**) and MBP-cleaved CHIP proteins (**C** and **D**). The reaction was incubated for 1 h at 37°C and samples were analyzed for both Hsc70- and self-ubiquitination by SDS/PAGE and immunoblotting using antibodies against Hsc70 and CHIP respectively. As a negative control, the WT CHIP protein was used in a separate reaction without adding ubiquitin.

CHIP participates in the ubiquitination of unfolded or misfolded substrates bound to chaperones via its U-box domain. In addition to the substrates, CHIP itself, and the heat shock protein chaperones are also known to be ubiquitinated by CHIP. To examine the enzymatic activity of the variants, ubiquitination assays were performed on recombinant CHIP both as MBP-fusion (Figure 3A and B) and as MBP-free (cleaved) proteins (Figure 3C and D). We investigated both the ability to ubiquitinate its substrate Hsc70 (Figure 3A and C) and its self-ubiquitination capacity (Figure 3B and D).

As seen from Figure 3(A) and (C), N65S and T246M displayed impaired Hsc70-ubiquitination activity for both MBP-fusion and MBP-free forms. Hsc70 seemed to be mono-ubiquitinated by the N65S mutant, while no Hsc70-ubiquitination was detected for T246M, confirming previous findings on these two variants [3,9]. Furthermore, T246M was unable to self-ubiquitinate both as MBP-fusion and cleaved proteins, while the N65S mutant showed similar level of self-ubiquitination as the WT (Figure 3B and D). The ubiquitination activities of the other mutants (E28K, K145Q, M211I, and S236T) were not overtly different from WT CHIP.

### Protein stability analysis by limited trypsin proteolysis

**Figure 4:**
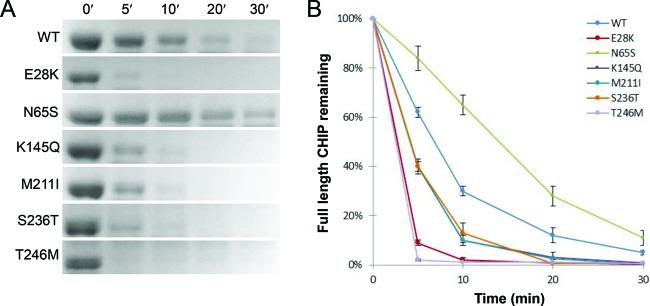
Limited proteolysis by trypsin of WT and mutant CHIP proteins The proteolytic susceptibility of MBP-free CHIP proteins was explored for WT and mutants using trypsin for protein digestion. (**A**) Proteins were detected by SDS/PAGE and SYBRO Ruby staining after being subjected to trypsin proteolysis for various time periods (0–30 min). (**B**) Full-length proteins were quantified by ImageJ software, and the data for the average of three individual experiments were plotted against time. Each time point represents the mean of three readings conducted on three separate days ± SD (*n*=3).

In order to elucidate the protein structure and folding of the mutants, limited proteolysis assay was performed using trypsin digestion (Figure 4). Compared with WT, increased rate of trypsin degradation was demonstrated for all variants except N65S, with E28K and T246M being mostly affected. Interestingly, N65S presented a decreased trypsin-degradation rate compared with WT, indicating that the N65S mutation leads to a more compact and stably folded protein structure where the trypsin cleavage sites are most likely less accessible.

### Oligomeric structures of MBP-CHIP fusion proteins analyzed by size-exclusion chromatography

**Figure 5:**
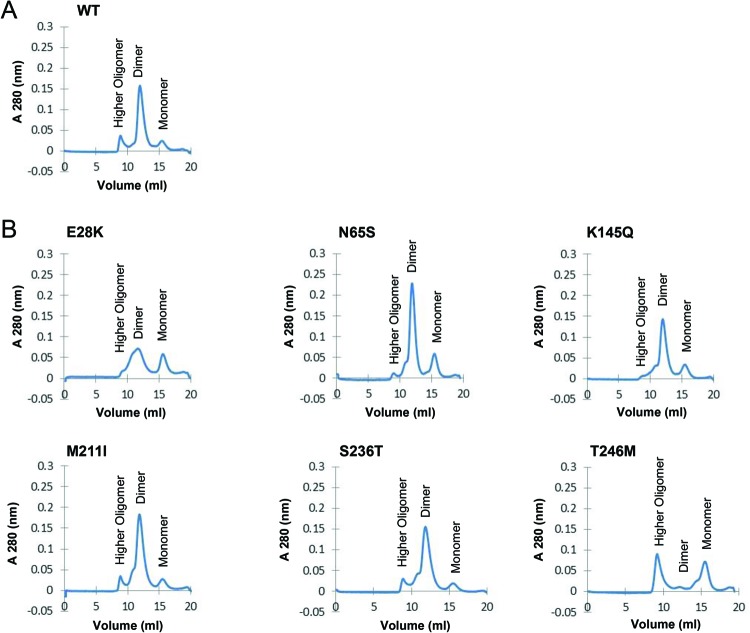
SEC of mutant recombinant MBP-CHIP fusion proteins All the experiments were performed on 1 mg of MBP-CHIP at 0.3 ml/min at 4°C, using Superdex 200 10/100 GL columns and phosphate buffer (20 mM NaH_2_PO_4_, pH 7.4) as the running buffer, while monitoring protein absorbance at 280 nm. The elution profile of fractions corresponding to monomers, dimers, and higher oligomers are shown for the (**A**) WT CHIP and (**B**) mutants E28K, N65S, K145Q, M211I, S236T, and T246M.

The effects of mutations on the oligomeric structures of the MBP-CHIP proteins were studied by size-exclusion chromatography (SEC) (Figure 5). As expected, WT MBP-CHIP exists mainly in a dimeric form. Other high multimeric forms and monomeric WT MBP-CHIP could also be detected by SEC, although these forms were of low abundance (Figure 5A).

In the chromatogram of E28K, the peak representing dimeric CHIP was less pronounced compared with WT CHIP with relatively more monomers and possibly tetramers (Figure 5B). In contrast, for the N65S mutant, the results indicate large amounts of dimers and less high-oligomeric forms compared with WT. The chromatograms for K145Q, M211I, and S236T were similar to WT. Finally, gel-filtration of the T246M mutant showed high levels of large oligomeric structures as well as monomers, with very little dimeric CHIP. Altogether, these data suggest that the oligomeric structures of MBP-CHIP fusion protein are most prominently affected by mutations E28K and T246M, demonstrated by reduced dimeric peaks (E28K and T246M) and higher oligomer peaks (T246M).

### Oligomeric states of MBP-CHIP fusion proteins analyzed using native polyacrylamide gel electrophoresis

**Figure 6:**
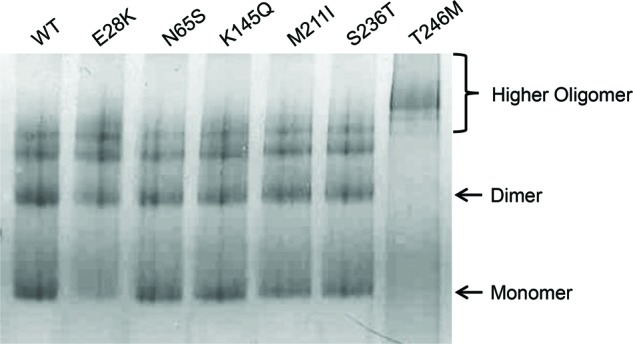
Oligomeric states of the WT and mutant MBP-CHIP by native gel electrophoresis Ten micrograms of WT and mutant MBP fusion protein was tested for migration on a 12% native-PAGE gel. Protein bands were visualized following Coomassie Blue staining.

Native polyacrylamide gel electrophoresis (native-PAGE) allows for a high-resolution analysis of the oligomeric state and molecular mass of native protein structure by using Coomassie G-250 as a charge-shift molecule. Separation of five major protein bands was observed for all the samples except for T246M (Figure 6). T246M migrated as a higher-order oligomer form, suggesting formation of aggregates for this mutant. The E28K mutant exhibited lower amounts of monomers (reduced by 67%), as seen by a faint monomer band in addition to lower amounts of dimers (reduced by 28%), compared with the WT, indicating a small shift toward the lower-order in the dimer–oligomer equilibrium for this mutant. Overall, these results suggest that while the higher-order oligomers serve as the major conformational state of T246M, the other mutants displayed almost the same oligomeric patterns as WT MBP-CHIP with small deviations observed for the E28K variant.

### Secondary structure content of MBP-CHIP fusion proteins measured by circular dichroism spectroscopy

**Figure 7:**
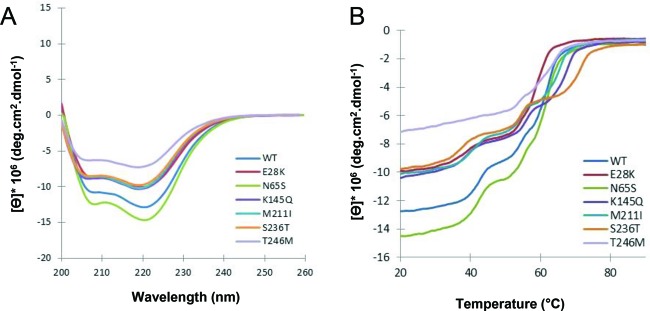
Secondary structure content and thermal unfolding profiles of WT and mutant CHIP proteins (**A**) Far-UV CD spectra were recorded for 6 µM WT and mutant MBP-CHIP proteins in the range of 200–260 nm at 20°C. (**B**) Thermal unfolding curves were obtained for WT and mutant MBP-CHIP proteins by monitoring CD signal at 222 nm from 20 to 90°C. All the spectra presented in this figure are background corrected and smoothed.

Crystallographic analysis of mouse CHIP indicates high degrees of α-helical structure due to the presence of two domains consisting mainly of α-helices (TPR and CC domains) [17]. Results from the far-UV circular dichroism (CD) spectra of both WT and mutant MBP fusion proteins demonstrated two minima at 208 and 222 nm, which is typical of proteins with a high helical content (Figure 7A). The difference in ellipticity of CD signals ([Ɵ]) observed for the mutants indicates changes of the conformational properties of these proteins relative to WT. Minor changes were observed for E28K, K145Q, M211I, and S236T, whereas a large loss of α-helicity was noticed for T246M. In contrast, N65S was identified as the only mutant with increased α-helical content compared with the WT.

### Thermal unfolding of MBP-CHIP fusion proteins as measured by circular dichroism

The effect of mutations on the thermal stability of MBP-CHIP protein was examined by investigating molar ellipticity (Ɵ) at 222 nm as a function of temperature (Figure 7B). The mid-point of each of the three transitions (mid-point of the thermal denaturation,* T*_m_) for denaturation is given in Table 1 and was determined from the first derivatives of the transition curve. Transition temperatures in the range of ∼41–45°C, ∼55–57°C, and ∼62–74°C were observed for denaturation of all the protein samples, except for T246M with only two transitions at ∼55 and 67°C (Figure 7B, light purple line and Table 1). This mutant also demonstrated a decrease in ellipticity, indicating loss of secondary structure. The loss in ellipticity, in addition to a more gradual unfolding with an apparent loss of the first transition seen for WT and all other mutants, indicates an unstable protein structure more prone to aggregation. The other mutants presented unfolding profiles with similar shapes as compared with the WT curve, although with smaller changes in the transition temperatures. For comparison, the thermal denaturation of MBP protein alone monitored by CD (Supplementary Figure S2) exhibited one *T*_m_ value of 56°C. This transition temperature corresponds well with the second transition observed for the MBP-fusion protein samples (Table 1). Therefore, it is most likely that the first (∼41–45°C) and third transitions (∼62–74°C) of the thermal denaturation curves of MBP-CHIP proteins are associated with unfolding of the CHIP protein itself and not MBP. We can only speculate that the first transition represents denaturation of the dimeric CHIP, while the third transition represents unfolding of the monomeric CHIP. Comparing the *T*_m_ of ∼44°C (first transition) seen for WT MBP-CHIP, all of the mutants showed lower first transitions *T*_m_ values than WT (Table 1), indicating decreased thermal stability of dimeric structures for the mutants. Hence, S236T with the first transition *T*_m_ of ∼41°C was regarded as the least stable protein in the dimeric conformation (Figure 7B, orange line and Table 1). Moreover, the absence of first transition on the thermal denaturation curve of T246M (Figure 7B, light purple line) suggested a very small amount of dimeric structures for this protein. The apparent *T*_m_ of CHIP mutants varied more in the third transition (possible monomeric phase) than the first transition (possible dimeric phase), as can be seen by the melting transitions ranging from ∼62 to 74°C in the third transition compared with ranging from 41 to 45°C in the first transition (Table 1).

**Table 1:** Mid-point of transitions for thermal unfolding of MBP-CHIP proteins

*T*_m_	WT	E28K	N65S	K145Q	M211I	S236T	T246M
***T*_m_ (1)**	44.2	42.4	44.5	42.7	43.9	41.2	–
***T*_m_ (2)**	55.8	55	56.2	57	56.2	56.2	55.4
***T_m_* (3)**	64.5	62.4	65.1	69.5	67.7	73.8	66.8

The transitions temperatures (°C) were determined from the first derivative of the thermal unfolding curves using the Spectra Manager software (Jasco products).

## Discussion

The present study provides additional insight concerning the effect of *STUB1* mutations in the development of ARCA disease through examining the activity and structural properties of the encoded CHIP protein mutants. Our results illustrate that some *STUB1* mutations known to cause recessive SCAR16 can affect the protein structure and stability in addition to the ability of CHIP to dimerize *in vitro.* This provides an alternative mechanism to the previously suggested direct effects on CHIP’s E3 ubiquitin ligase properties and interaction with its chaperones [3]. We speculate that at least some *STUB1* mutations mediate disease by affecting the CHIP E3 ubiquitin ligase interactions and function through modification of its oligomeric states and structural stability. As a result, the protein quality control system fails to eliminate damaged proteins properly which, in turn, cause toxicity and cell death.

### The effects of different *STUB1* mutations on the E3 ubiquitin ligase activity of CHIP

Reduced levels of Hsc70-ubiquitination activity were observed for N65S located in the TPR domain. This mutation affects an asparagine residue that was previously reported to be involved in CHIP substrate binding [18]. However, another mutation in the same domain (E28K) resulted in similar ubiquitination activity as the WT for both Hsc70 substrate- and auto-ubiquitination, indicating that the substrate binding of CHIP was not affected by this mutation. Complete loss of ubiquitination activity was observed for CHIP-T246M for both Hsc70 substrate and CHIP itself. The T246M substitution (located at the U-box domain of CHIP) can affect the interaction of CHIP with the ubiquitin–proteasome pathway components and result in abolished ubiquitination activity of the CHIP protein as also suggested by Shi et al. [9]. These findings are in complete accordance with previous results obtained from the ubiquitination activity analysis of these three mutations [3,9].

The ubiquitination activity of the three variants K145Q, M211I, and S236T has to our knowledge not been investigated before, and our results revealed that they have WT-like ubiquitination activities *in vitro* (Figure 3). Interestingly, M211I and S236T are not found in the Exome Aggregation Consortium (ExAC) and K145Q only in heterozygous carriers (Minor allele frequency = 0.07%). All three variants affect highly conserved amino acid residues (Figure 1C) and all were reported to co-segregate with the disease [4,7,10]. Altogether, this indicates that these missense variants are pathogenic, but do not affect the ubiquitination activity *in vitro*. Furthermore, it was reported that CHIP protein levels are reduced in HEK293 cells transfected with M211I [4], supporting a loss-of-function mechanism. These three mutations, together with E28K, have been found in ARCA patients in a compound heterozygous form together with either a non-sense or a frameshift *STUB1* mutation [3,5,7,10]. The premature stop codon in the second allele probably leads to the truncation of the protein and/or to nonsense-mediated RNA decay.

### Oligomerization studies discovered high aggregation propensity for T246M

SEC showed that T246M forms aggregates, pointing to a conformational change in the structure of this mutant (Figure 5), and further confirmed by native-PAGE (Figure 6) and in corroboration with others [19]. The necessity of dimerization for activity of CHIP was indicated by a study on CHIP from *Homo sapiens* (hCHIP), where a deletion mutant lacking the central CC domain, important for CHIP dimerization, was shown to have no E3 ligase activity [15]. Another study analyzing the crystal structure of Prp19 U-box suggested dimerization as a common architecture for the U-box and RING-finger families of E3 ubiquitin ligases, which play an essential role in the stability and functionality of these enzymes [20]. Thus, the complete loss of function observed in the *in vitro* ubiquitination activity assay for the T246M mutant might be caused by both impaired ubiquitin ligase activity (as a result of impaired interaction with ubiquitination enzymes) and formation of oligomeric protein structures. On the contrary, increased amount of dimeric structures was observed for N65S with a dimer peak higher than that of the WT (Figure 5B). However, since the mutation is not located in the CC domain of CHIP, which is important for dimerization, the mutation might indirectly lead to general conformational changes that promote CHIP dimerization.

### Mutants T246M and N65S displayed remarkable differences in secondary structure content

The α-helical content of WT CHIP obtained from its far-UV CD spectroscopy spectrum corresponds well with the previously reported crystallographic data (Figure 7A)[17]. Reduced levels of ellipticity were, however, observed in the spectra from the majority of mutants, suggesting decreased levels of α-helical structure content in these mutants compared with WT. The largest loss of α-helicity was shown for the T246M mutant, which can be explained by its tendency to aggregate as discussed above. On the contrary, the N65S mutation generated a CHIP structure with an increased secondary structure content of α-helices, and corroborated the SEC findings for this variant (Figure 5B).

It is important to consider limitations associated with the use of MBP fusion proteins while monitoring structural properties. The remarkable ability of MBP to increase the solubility of fusion partners has made it a commonly used affinity tag for protein purification. Unfortunately, we were not able to purify CHIP from MBP despite several attempts using both amylose and nickel affinity chromatography, and SEC (data not shown). The latter could probably be explained by the similar molecular weights of CHIP and MBP of 35 and 43 kDa respectively. Therefore, we cannot be certain whether the overall protein folding and assembly may be different for MBP-CHIP fusion proteins compared with purified MBP-free CHIP. It is, therefore, possible that our assay gives a conservative estimate of the mutational effects on misfolding and aggregation due to the potential chaperone assisting properties of MBP [21]. However, as the CD results of WT CHIP are very comparable with two previous CD studies on purified MBP-free CHIP [15,22], it is probable that the MBP does not have substantial effects on the results in our assay.

In addition, since our main focus was to investigate differences in protein structure/folding of WT CHIP compared with CHIP mutants, rather than the folding of WT CHIP per se, we believe our structural analyses of MBP fused to CHIP are relevant.

### N65S, the only mutant with increased structural stability against limited proteolysis

Limited proteolysis of a globular protein occurs mostly at flexible loops, and regular secondary structures such as helices are not subjected to cleavage (segmental mobility). Thus, the decreased susceptibility identified for CHIP-N65S against limited proteolysis is expected to be associated with a structure that contains a larger number of α-helices compared with the WT, which is in corroboration with the far-UV CD spectra of this mutant (Figures 4 and 7). The induced stability and proteolytic resistance in the structure of N65S may also be a consequence of more dimeric states discovered during gel-filtration analysis of this mutant (Figure 5B). In addition, a protein’s susceptibility to proteolysis can be functionally linked to its energy landscape that is encoded within the amino acid sequence [23]. With this view, proteolytic susceptibility of folded proteins requires access to high-energy cleavable sites, making a compactly folded protein a poor substrate for proteolytic digestion. Therefore, decreased susceptibility of N65S can be the result of acquiring a more compact structure compared with the WT and the other mutants.

Mutants K145Q, M211I, and S236T displayed approximately the same degree of proteolytic digestion, being lower than that of T246M yet higher compared with the WT. This suggests that the former proteins achieve a somewhat looser, flexible structure, which makes them more susceptible toward proteolysis. This is in agreement with the results from far-UV CD where these mutants all showed similar loss of ellipticity compared with the WT at equal protein concentrations, indicating a loss in secondary structure. An additional decrease in α-helical content in the structure of K145Q, M211I, and S236T mutants could explain their reduced susceptibility against limited proteolysis. A loss in ellipticity was also observed for E28K; however, this mutant presented high level of susceptibility toward limited proteolysis similar to that of T246M. An explanation might be the high amount of trimeric/tetrameric structures identified for this protein during gel-filtration analysis that could indicate that the high proteolytic susceptibility of E28K is due to the unfolded/flexible structures of the protein molecule that appear during the formation of lower-order oligomers [24].

### Circular dichroism revealed new insights into the conformational dynamics and thermal stability of CHIP protein mutants

Denaturation of small globular proteins generally follows a two-state mechanism involving a single unfolding transition (*T*_m_), and two forms of fully native (N) and unfolded (U) proteins. However, many proteins have recently been observed to stabilize intermediates between the N and U states, and therefore show more than one transition during their unfolding profile [25,26]. This behavior is usually found in multi-domain/multimeric proteins where different domains/meres unfold independently and at different temperatures. Analysis of data obtained from such curves is often more complicated than that of a single-stage unfolding pattern. In the case of MBP-CHIP, the three transitions detected during thermal denaturation of the WT protein are assumed to be associated with unfolding of (in order) dimeric CHIP, MBP, and monomeric CHIP (Figure 7B). Dimeric CHIP is stabilized by both inter- (between the monomers) and intramolecular (within the monomers) forces. Therefore, it is possible that the disruption of intermolecular interactions during the first transition temperature (∼44°C) of MBP-CHIP proteins generates both dissociated MBPs and monomeric CHIPs. Subsequently, increased temperature results in unfolding of each MBP and CHIP monomers through disruption of interactions within the proteins (intramolecular interactions). By examining the thermal denaturation of ‘MBP-only’ (Supplementary Figure S2), and based on other CD studies done on the unfolding transition of this protein [21,27], the second transition of MBP-CHIP (∼56°C) is interpreted to arise from MBP alone. Therefore, CHIP itself (monomer) is expected to unfold at ∼64°C corresponding to the last transition temperature.

All investigated mutants apart from T246M presented thermal unfolding curves similar to WT, indicating that the whole conformation and dynamics of the protein were not largely affected by the mutations. Rather, differences are more pronounced between the dimeric and monomeric unfolding transition temperatures that determine the thermal stability of mutant protein structures. In the dimeric state, N65S has similar stability as WT, but higher than other mutants (*T*_m_ of ∼44°C). The higher degree of dimerization observed during SEC analysis of this mutant can possibly explain this observation. Moreover, N65S was the only mutant with an increased level of α-helicity and stability against limited proteolysis. Taken together, these findings indicate a stabilizing effect for N65S mutation, resulting in a more compact CHIP protein structure.

To summarize our data, we can conclude that the ubiquitination activity of CHIP was impaired under the effect of mutations N65S and T246M while the other mutants showed intact activities. Increased amounts of dimeric structures, as well as higher levels of secondary structures, were discovered for N65S. In contrast, the T246M mutation generated a flexible protein structure with decreased α-helicity and a high tendency for aggregation. Decreased secondary structure content and proteolytic stability was observed for K145Q, M211I, and S236T. Overall, these results provide first *in vitro* evidence regarding the protein structural and folding properties, as well as new additions on the ubiquitination activity of *STUB1* mutations. Future studies should focus on the *in cellulo* characterization of mutations, using appropriate cell lines and animal models. Since all the mutants were associated with altered stability *in vitro*, it is likely that this will lead to reduced cellular levels of these variants, as was seen for E28K, N65S, and M211I previously [3,4]. Therefore, further investigations are required in order to find out how misfolding of CHIP itself can lead to the development of SCAR16 *in vivo*.

## Accession numbers

*STUB1* cDNA: GeneBank accession no. **NM_005861.3**

## Abbreviations

ARCA: autosomal recessive cerebellar ataxia
CC: coiled-coil
CD: circular dichroism
CHIP: C-terminus of Hsc70-interacting protein
ExAC: Exome Aggregation Consortium
Hsc: Heat shock cognate
Hsp: Heat shock protein
MBP: maltose-binding protein
PDB: Protein Data Bank
SCAR: Spinocerebellar ataxia, autosomal recessive 16
SCAR16: spinocerebellar ataxia 16
SDS/PAGE: sodium dodecyl sulfate/polyacrylamide gel electrophoresis
SEC: size-exclusion chromatography
STUB1: STIP1 homology and U-box containing protein 1
TEV: Tobacco etch virus
*T*_m_: mid-point of the thermal denaturation
TPR: tetratricopeptide repeat
WES: whole exome sequencing
WT: wild-type
